# Acland's Practice Manual for Microvascular Surgery: Third Edition 2008

**Published:** 2008

**Authors:** Mukund R. Thatte

**Affiliations:** Consultant Hand and Plastic Surgeon, Prof. of Plastic Surgery, Bombay Hospital Institute of Medical Sciences, Editor, IJPS, Mumbai, India. Email: mthatte@vsnl.com

It is always a great pleasure to review a book by one of our living legends viz. Dr. Robert D. Acland. This time he is joined by Dr S. Rajasabapathy - a very well known and respected proponent of the art in this country. The reason for the third edition and the changes are well described by Dr. Acland in the preface. The 2nd edition was published in 1989 and in the present one they have pruned many topics which would not be of much use today or are well known and understood (like the suppliers of microscopes, sutures, etc.). In addition, the authors have taken efforts redoing and refining many illustrations. The book considered as the bible for the micro trainees had only things related to the micro course in the lab in its earlier avatars. It was always felt that there must be information to make the transition from the lab to the clinical setting and this has been made good in the third edition. A good attempt has been made to answer all the doubts that come up in the minds of the young surgeon venturing into the field. Questions that were asked in the meetings by trainees were collected and answered (personal communication from Dr S. Rajasabapathy). While there may not be one single method for doing a particular thing, the authors have made a choice and have described a safe method, which is I think very valuable for the new surgeon in the field. The format is slightly different. The book is now spiral bound - so that the pages could be open if the trainee uses it in the lab and for easy reading.

Dr. Acland has very graciously transferred the copyright to the ISSH and the proceeds go to the establishment of an overseas travelling fellowship of the ISSH, which will be useful to further the Indian participation in hand and microsurgery around the globe. I would recommend every trainee and practising microsurgeon to buy it - remember the proceeds are used to further your own education. We need to start a virtuous cycle.

How is it available?

In India it is available at a price of Rs. 500 (from ISSH) and M/s S and T have very kindly taken up the responsibility to make it available in the rest of the world. For further information contact info@s-and-t.net

